# Low serum calcium: a new, important indicator of COVID-19 patients from mild/moderate to severe/critical

**DOI:** 10.1042/BSR20202690

**Published:** 2020-12-22

**Authors:** Xi Zhou, Dong Chen, Lan Wang, Yuanyuan Zhao, Lai Wei, Zhishui Chen, Bo Yang

**Affiliations:** 1 Institute of Organ Transplantation, Tongji Hospital, Tongji Medical College, Huazhong University of Science and Technology, Wuhan, Hubei, China; 2 Key Laboratory of Organ Transplantation, Ministry of Education, Ministry of Public Health, Chinese Academy of Medical Sciences, Wuhan, Hubei, China; 3 Reproductive Medicine Center, Tongji Hospital, Tongji Medicine College, Huazhong University of Science and Technology, Wuhan, Hubei, China

**Keywords:** COVID-19, Multi-organ injury, Pro-inflammatory cytokine, Serum calcium

## Abstract

**Background:** Coronavirus disease 2019 (COVID-19) virus is still spreading, finding out the initial hits of viral infection is important to minimize the mild/moderate population, prevent disease aggravation and organs dysfunction.

**Objective:** We investigated COVID-19 patients with different serum calcium levels.

**Design:** We checked the serum calcium level of the patients based on days after symptom onset as well as the severity of COVID-19. We also checked multiorgan injuries and immune cytokines level in their blood.

**Results:** Both mild/moderate and severe critical cases we observed showed low calcium level in the early stage of viral infection, while the severe/critical cases showed significant lower calcium level than mild/moderate cases in the early stage. We also found that low calcium level related to severe/critical multiorgan injuries especially in the mild/moderate population. Proinflammatory cytokine IL-6 also correlated to calcium change in both mild/moderate and severe/critical cases.

**Conclusions:** Our finding indicates that calcium balance is a primal hit of COVID-19 and a biomarker of clinical severity at the beginning of symptom onset. Calcium is closely associated with virus-associated multiple organ injuries and the increase in inflammatory cytokines. Our results provide a new, important indicator of COVID-19 patients from mild/moderate to severe/critical: serum calcium.

## Introduction

Coronavirus disease 2019 (COVID-19) caused by severe acute respiratory syndrome coronavirus 2 (SARS-CoV-2) has spread globally [1,2]. As of 16 October 2020, a total of 39023292 confirmed cases and 1099586 deaths have been reported in more than 200 countries worldwide [3]. While 80% of COVID-19 infections have less severe clinical symptoms (low-grade fever, cough, fatigue) with no evidence of pneumonia or even asymptomatic, those mild or moderate cases are still contagious and move toward severe/critical [4]. Once the mild/moderate case becomes severe/critical, it is always accompanied by pneumonia and multiorgan injuries [5]. As the virus is still spreading, finding out the initial hits of viral infection is important to minimize the mild/moderate population, prevent disease aggravation and organs dysfunction.

A report showed that 70% of acutely ill patients had decreased levels of serum calcium which is associated with a poor prognosis [6]. Previous studies of COVID-19 patients showed that severe/critical cases with electrolyte disturbance including calcium unbalance [7,8], hypokalemia in COVID-19 patients had been associated with ECG changes including long QT [9,10]. But the role of abnormal calcium level in COVID-19 is unknown.

## Materials and methods

### Study design and patients

This single-center, retrospective, observational study was approved by the Institutional Review Board (IRB) at Tongji Hospital, Tongji Medical College, Huazhong University of Science and Technology (IRB approval number: TJ-IRB20200364). The written informed consent was waived by our IRB since this was a retrospective study that assessed de-identified data and included no potential risk to patients. From 29 January to 10 February 2020, 127 patients with confirmed COVID-19 admitted to Tongji Hospital were enrolled in this retrospective study.

### Diagnosis and classification

All patients were definitely diagnosed with pneumonia by chest CT scans. The throat swab samples were tested by reversing real-time polymerase chain reaction (RT-PCR) to detect the presence of SARS-CoV-2 [11]. A definitive diagnosis of COVID-19 pneumonia was made based on the following two criteria: (1) radiographic evidence of pneumonia, and (2) positivity of respiratory specimens for SARS-CoV-2 by RT-PCR assay. A clinical diagnosis of COVID-19 pneumonia was made on the basis of symptoms, exposure(s) and presence of lung imaging features consistent with coronavirus pneumonia [12].

According to the New Coronavirus Pneumonia Diagnosis and Treatment Program (7th edition) published by the National Health Commission of China, clinical classification of COVID-19 pneumonia mainly includes mild (i.e. minor clinical symptoms (e.g. fever, cough) without imaging manifestations), moderate (i.e. fever or respiratory tract infection symptoms with imaging indicating pneumonia), severe (i.e. dyspnea, respiratory frequency ≥ 30/min, blood oxygen saturation ≤ 93%, partial pressure of arterial oxygen-to-fraction of inspired oxygen ratio < 300 and/or lung infiltrates > 50% within 24–48 h), and critical (i.e. respiratory failure, septic shock and/or multiple organ dysfunction or failure) [12,13]. We combined mild and moderate patients into one group (mild/moderate) and another group (severe/critical) included severe and critical patients.

### Data collection

The baseline clinical characteristics, including age, underlying disease, days from onset to admission, and clinical classifications were collected from electronic medical records, and all laboratory tests were performed according to the clinical needs of patients. The levels of electrolyte (Natrium: Na, Kalium: Ka, Chlorine: Cl, Calcium: Ca), highly sensitive cTnI (hs-cTnI), N-terminal pro b-type natriuretic peptide (NT-proBNP), blood urea nitrogen (BUN), creatinine (CRE), estimated glomerular filtration rate (eGFR), alanine aminotransferase (ALT), aspartate aminotransferase (AST), total bilirubin (TBIL) and direct bilirubin (DBIL) measured by Roche COBAS8000 were recorded. Prothrombin time (PT) were measured by Stago STA-R EVOLUTION and the worst SpO2 within 24 h of admission were measured by ABL800. Moreover, the cytokine levels of 93 patients were tested according to the clinical needs of patients. All the blood parameters were assayed by clinical laboratory of Tongji Hospital.

### Statistical analysis

In our retrospective statistical analysis, for continuous data, D’Agostino and Pearson normality test and Shapiro–Wilk normality test were used to test if the data had normal distribution. For normal distribution, One-way ANOVA or Two-way ANOVA were applied. For data not normally distributed, either non-parametric Mann–Whitney test (two-tailed) or Kruskal–Wallis test was used, data were expressed as Line at median and minimum–maximum were represented by the top/bottom of violin. Continuous data analyses were performed using GraphPad Prism 7. The χ^2^ test measured differences between categorical variables, and the Fisher’s exact test was used when the data were limited. A *P*-value of less than 0.05 was considered to be statistically significant with a two-sided alternative. Categorical variables analyses were performed using SPSS version 23.0 (SPSS Inc., Chicago, IL).

## Results

**Figure 1:**
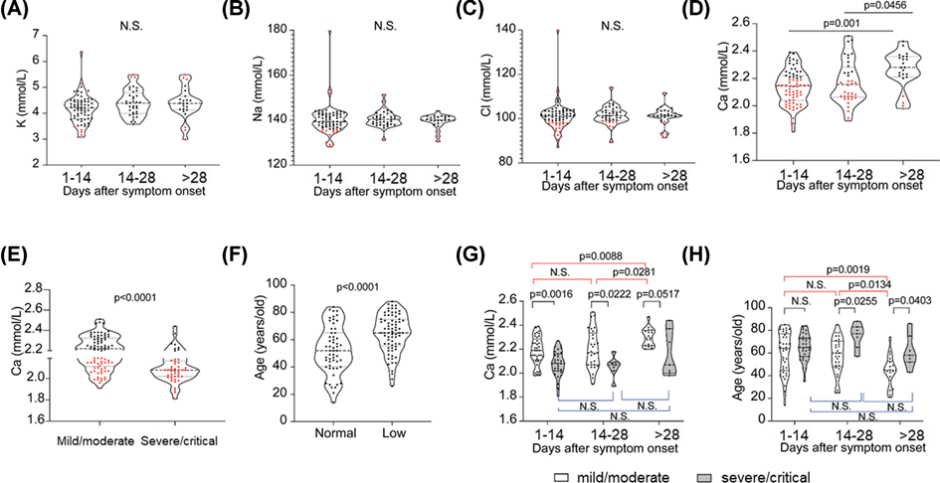
The association between serum calcium in COVID-19 patients with disease severity, age and days after symptom onset Independent group *t* test was used to compare the means for continuous variables. A *P*-value of less than 0.05 was considered to be statistically significant with a two-sided alternative. (**A–D**) Various kinds of serum ions (a. potassium ion; b. sodium ion; c. chloride ion; d. calcium ion) concentrations in COVID-19 patients vary with the number of days after symptom onset. (**E**) Calcium level in COVID-19 patients grouped by severity of illness. (**F**) Low calcium and normal patients age distribution (normal (serum calcium concentration ≥ 2.2 mmol/l), low (serum calcium concentration < 2.2 mmol/l). (**G**) Calcium level in mild/moderate and severe/critical COVID-19 patients vary with the number of days after symptom onset. (**H**) The age distribution of mild/moderate and severe/critical cases in different timing after symptom onset was checked.

In the present study, we collected 127 COVID-19 patients and tracked their blood laboratory test report, we found a major electrolyte disturbance after symptom onset was calcium changing (Figure 1A–D). Based on the New Coronavirus Pneumonia Diagnosis and Treatment Program (7th edition) published by the National Health Commission of China, we separated all patients into two groups: mild/moderate and severe/critical cases. We compared the calcium level regardless of the time of symptom onset, 80.9% severe/critical patients had calcium level below 2.2 mmol/l and it was lower than mild/moderate group (*P*<lt0.0001) (Figure 1E), and 41.0% of mild/moderate patients also showed calcium level under 2.2 mmol/l (Figure 1E). This result suggested that low calcium is a common abnormal parameter in COVID-19 patients regardless of the severity, if we donot consider the time of viral infection. Thus, we could not simply use calcium as a biomarker to define the clinical severity of COVID-19. We further analyzed the calcium change in different clinical severity groups after symptom onset (Figure 1G): in the mild/moderate group, the calcium level of COVID-19 disease were significantly lower (*P*=0.0088) in the early stage (1–28 days) than later stage (after 28 days) which showed normal calcium level at that point in time, but in the severe/critical group there was only a bit alleviation after 28 days, the severe/critical patients always presented lower calcium level than mild/moderate group in the early stage (1–28 days).

As we had seen that the elder had lower calcium level in blood serum (Figure 1F) as they tended to have osteoporosis and poor ability of calcium absorbing, we wondered that if age associated with low calcium lead to the difference of calcium level in COVID-19 patients, so the age distribution of mild/moderate and severe/critical cases in different timing after symptom onset was checked (Figure 1H). In the very early stage of COVID-19 there was no difference of age between mild/moderate and severe/critical cases, but they showed different proportion of low calcium (54.2 vs 83.9%) (Figure 1H). The mild/moderate patients who showed normal calcium level in the late stage of COVID-19 were younger than the same group cases who showed lower calcium level in the early stage of viral infection (Figure 1H).

**Figure 2:**
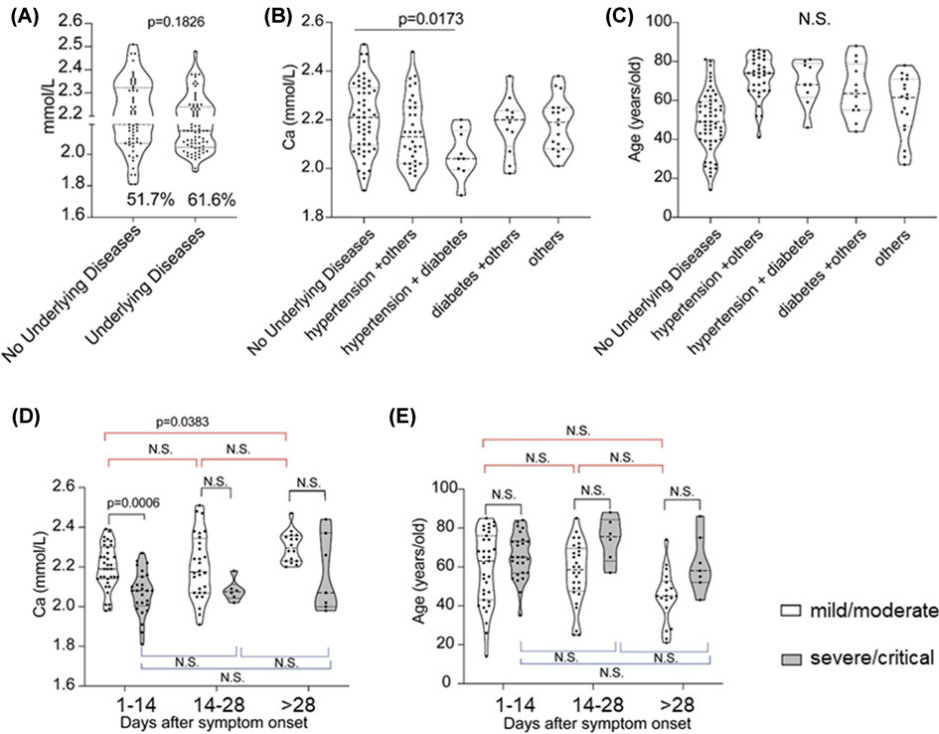
The calcium level in the patients with or without coexisting disorder (**A**) The calcium level in the patients with or without coexisting disorder. (**B,C**) Patients were divided into five groups (no underlying diseases, hypertension+others, hypertension+diabetes, diabetes+others, others) and measured the calcium level (B) and the age distribution (C) separately for each group. (**D,E**) When we exclude the patients with hypertension plus with diabetes, the calcium level in mild/moderate and severe/critical COVID-19 patients vary with the number of days after symptom onset (D) and the age distribution of mild/moderate and severe/critical cases in different timing after symptom onset was checked (E).

Recent Center for Disease Control and Prevention (CDC) data on hospitalized patients in 14 U.S. states found that approximately one-third had chronic lung disease—but nearly as many had diabetes, and fully half had pre-existing high blood pressure [14]. We also analyzed the calcium level in the patients with or without coexisting disorder (Figure 2A); we found the patients with hypertension plus with diabetes showed significantly lower calcium level than patients with no underlying diseases, and this abnormal datum was not relative with age difference (Figure 2B,C). Generally, there was no significant difference between patients with and without coexisting disorder, so we did not exclude the patients with hypertension plus with diabetes, even though we excluded those patients it did not affect our conclusion (Figure 2D,E).

As was reported, the virus attacked multiple organs of the body, especially in roughly 5% of patients who had become critically ill [15]. COVID-19 not only hit the lung with enriched angiotensin-converting enzyme 2 (ACE2) receptors but also entered heart lining blood vessels which also expressed ACE2 [16], while rectal and liver injury was also very common as kidney and liver were also enriched with ACE2 [17,18]. We selected hs-cTnI, NT-proBNP, BUN, CRE, eGFR, ALT, AST, TBIL, DBIL, PT and SpO2 as clinical parameters to show the injury level of multiple organs. We found the low calcium level significantly relative with severe organ injuries (Supplementary Table S1), while in the mild/moderate and severe/critical population different calcium level also affected organ injury severity (Table 1). The mild/moderate cases with low calcium also showed significantly lower NT-proBNP, eGFR and SpO2 than the mild/moderate cases with normal calcium; while in the severe/critical cases with low calcium showed the significantly low level of hs-cTnI and PT, the eGFR level was close to being statistically significant (*P*=0.055), and AST was just about significant (*P*=0.051). NT-proBNP and SpO2 were too worse in the severe/critical population with either normal or low blood calcium, so there was no remarkable significance (*P*=0.231 and 0.272).

**Table 1:** Serological indicators of multiple organ injury in mild/moderate and severe/critical patients stratified by serum calcium

	Mild/Moderate			Severe/Critical		
	Normal Ca	Low Ca	P-value	Normal Ca	Low Ca	P-value
*n*	46	36		8	37	
hs-cTnI (>15.6 pg/ml)	3/46 (7.1%)	4/36 (12.9%)	0.694^*^	0/8 (0.0%)	19/37 (54.3%)	0.014^*^
NT-proBNP (>169 pg/ml)	6/46 (14.3%)	12/36 (37.5%)	0.015	4/8 (50.0%)	23/37 (69.7%)	0.231^*^
BUN (>7.5 mmol/l)	2/46 (4.4%)	3/36 (8.3%)	0.650^*^	0/8 (0.0%)	12/37 (32.4%)	0.087^*^
CRE (>84 μmol/l)	6/46 (13.0%)	6/36 (16.7%)	0.645	2/8 (25.0%)	15/37 (40.5%)	0.690^*^
eGFR (<90 ml/min/1.73)	12/46 (26.1%)	20/36 (55.6%)	0.007	2/8 (25.0%)	24/37 (64.9%)	0.055^*^
ALT (>41 U/l)	13/46 (28.3%)	9/36 (25.0%)	0.741	1/8 (12.5%)	13/37 (35.1%)	0.402^*^
AST (>40 U/l)	8/46 (17.4%)	9/36 (25.0%)	0.399	1/8 (12.5%)	20/37 (54.1%)	0.051^*^
TBIL (>26 μmol/l)	0/46 (0.0%)	1/36 (2.8%)	0.255	0/8 (0.0%)	5/37 (13.5%)	0.568^*^
DBIL (>8 μmol/l)	2/46 (4.4%)	3/36 (8.3%)	0.454	0/8 (0.0%)	11/37 (29.7%)	0.169^*^
PT (>14.5 s)	5/46 (11.4%)	7/36 (19.4%)	0.276	2/8 (25.0%)	25/37 (71.4%)	0.045^*^
SpO_2_ (mean ± S.D.)	97.3 ± 1.4	96.0 ± 2.4	0.004	93.8 ± 3.9	89.5 ± 10.7	0.272

We applied the χ^2^ test to measure differences between variables, and the Fisher’s exact test was used when the data were limited (tagged with *). Independent group *t* test was used to compare the means for continuous variables. A *P*-value of less than 0.05 was considered to be statistically significant with a two-sided alternative. Levels of hs-cTnI, NT-proBNP, BUN, CRE, eGFR, ALT, AST, TBIL, DBIL, PT above the 99th percentile of the upper reference limit [hs-cTnI > 15.6 pg/ml, NT-proBNP > 169 pg/ml, BUN > 7.5 mmol/l, CRE > 84 μmol/l, eGFR < 90 ml/min/1.73, ALT > 41 U/l, AST > 40 U/l, TBIL > 26 μmol/l, DBIL > 8 μmol/l, PT > 14.5 s] on hospital admission.

We also compared immune cytokines level in blood in mild/moderate and severe/critical patients stratified by serum calcium. Only interleukin (IL) 6 (IL-6) as a key factor was activated in low calcium cases in both mild/moderate and severe/critical groups (*P*=0.004 in mild/moderate groups, *P*=0.037 in severe/critical groups; Table 2 and Supplementary Table S2).

**Table 2:** Immune cytokines level in blood in mild/moderate and severe/critical patients stratified by serum calcium

	Mild/Moderate			Severe/Critical		
	Normal Ca	Low Ca	*P*-value	Normal Ca	Low Ca	*P*-value
*n*	31	30		5	27	
IL-1β (>5 pg/ml)	3/31 (9.7%)	2/30 (6.7%)	>0.05	0/5 (0.0%)	3/27 (11.1%)	>0.05
IL-2R (>710 U/ml)	3/31 (9.7%)	7/30 (37.5%)	>0.05	1/5 (20.0%)	15/27 (55.6%)	>0.05
IL-6 (>7 pg/ml)	2/31 (6.5%)	11/30 (36.7%)	0.004	1/5 (20.0%)	20/27 (74.1%)	0.037
IL-8 (>62 pg/ml)	0/31 (0.0%)	0/30 (0.0%)	/	0/5 (0.0%)	7/27 (25.9%)	>0.05
IL-10 (>9.1 pg/ml)	2/31 (6.5%)	2/30 (6.7%)	>0.05	1/5 (20.0%)	16/27 (59.3%)	>0.05
TNF-α (>8.1 pg/ml)	9/31 (29.0%)	9/30 (0.3%)	>0.05	2/5 (40.0%)	17/27 (63.0%)	>0.05

Levels of IL-1β, IL-2R, IL-6, IL-8, IL-10, TNF-α above the 99th percentile of the upper reference limit [IL-1β > 5 pg/ml, IL-2R > 710 U/ml, IL-6 > 7 pg/ml, IL-8 > 62 pg/ml, IL-10 > 9.1 pg/ml, TNF-α > 8.1 pg/ml] on hospital admission. Abbreviations: IL-2R, interleukin-2 receptor; TNF-α, tumor necrosis factor-α.

## Discussion

In the present study, we analyzed the changes of different electrolytes after symptom onset. Although both serum potassium and serum calcium have been shown to decrease in COVID-19 patients (54% (95 of 175) of patients had serum potassium less than 3.5 mmol/l) [9]. A statistically significant lower serum calcium was noted in patients with severe COVID-19 (WMD: −0.20 mmol/l [95% CI: −0.25 to −0.20 mmol/l]) [7]), only serum calcium changed with the number of days of symptom onset. We checked the serum calcium level based on symptom onset days and also severity of COVID-19. We also checked multiorgan injuries and immune cytokines level in blood. Our study revealed that all cases regardless of the severity of their condition showed low calcium in the early stage of viral infection, the severe/critical case showed significant lower calcium level than mild/moderate cases in the early stage. We also found that in the later stage of COVID-19 the age associated with calcium imbalances affected the recovery ability of mild/moderate population, this is because as people get older, their liver and kidney functions decline, thus the serum level of 25-hydroxyvitamin D in human body decreases, leading to the intestinal calcium absorption disorder and thus affecting the recovery of blood calcium [19] Furthermore calcium was relative to multiorgan injuries, especially in severe/critical cases, so keeping calcium balance was so important to maintain normal organ functions. Proinflammatory cytokine IL-6 was also corelative to calcium changing in both mild/moderate and severe/critical cases.

How to prevent mild/moderate cases from turning into severe/critical illness is one of the key factors in COVID-19 prevention and treatment. Finding people who are prone to severe/critical illness can help to intervene in advance and reduce the conversion rate from mild/moderate into severe/critical illness. According to the New Coronavirus Pneumonia Diagnosis and Treatment Program (7th edition) published by the National Health Commission of China, there were four severe/critical warning indexes, including peripheral blood lymphocytes progressively decreasing, peripheral blood inflammatory cytokines (such as IL-6 and C-reactive protein) gradually increasing, lactic acid processivity increasing, and pulmonary lesions rapidly evolving in a short period of time [12]. This study suggested that while in the very beginning of viral infection, the calcium level could be a biomarker to evaluate the severity of COVID-19. It might help us at the early stage to predict which COVID-19 patient would be easily tended to severe/critical, we should give more attention and special care when sending those patients to self-quarantine at home.

However, until now the mechanism of calcium dysregulation is unclear. Many viruses have particular nonstructural proteins which can regulate the expression of host cell ion channel proteins called vasopressin, which defect the host’s normal ion homeostasis to promote viral replication and cause pathological damage. For example, rotavirus nonstructural protein 4 (NSP4) elevates cytosolic calcium ion by activating both uptake of extracellular calcium ion and release of ER calcium ion pools [20]. Elevated cytosolic calcium ion is directly related to the typical symptoms of rotavirus-induced diarrhea and vomiting [21]. Diarrhea and vomiting are also present in some COVID-19 patients, whether these symptoms are associated with hypocalcemia like in rotavirus needs more experiments to prove. Experiments have shown that SARS-CoV E gene encodes a small transmememium protein with a permeable calcium channel, leading to the entry of extracellular calcium ions into the cell. As several reports showed COVID-19 disturbed the immune system then caused cytokine storm [22]. We also compared cytokine level with different calcium levels. Intriguingly, only IL-6 as a key factor that triggered the cytokine storm in COVID-19 patients [23] was activated in low calcium cases in both mild/moderate and severe/critical groups. Changes in intracellular calcium homeostasis can promote the activation of inflammatory pathways, leading to the increase in inflammatory factors such as IL-1β, tumor necrosis factor (TNF) and IL-6 [24,25]. Given the high similarity between the SARS-CoV and SARS-CoV-2 genomes, the mechanism found in SARS-CoV-2 may be similar to SARS-CoV. So, whether COVID-19 hypocalcemia is due to the fact that certain proteins alter the calcium channels of host cells, thereby allowing extracellular calcium ions to enter the intracelluar needs further study. Other studies have shown that IL-6 can reduce serum calcium concentration by up-regulation of calcium-sensing receptor gene (CASR) [26]. We can speculate that low calcium levels in COVID-19 patients may be due to viral associated cytokine storm. IL-6 inhibitors can be used to inhibit the effect of IL-6 on serum calcium and avoid the damage of low calcium to multiple organs.

So far, it is unknown whether it is because the virus directly attacks the cells in different organs, then causes injury of organs, or because of lungs damage that leads to low oxygen which also destroys blood vessels, or a cytokine storm could ravage the heart as it does to other organs. Serum calcium plays a vital role in a number of important physiological functions including deposition of calcium phosphate, manipulation, neuron electrical signal transmission, hormonal regulation, and the blood coagulation [27]. Some studies indicate that serum calcium imbalance is associated with many organ injuries, for example, serum hypocalcemia was independently associated with all-cause mortality in critically ill patients with acute kidney injury (AKI) [28]. Low serum calcium was independent predictor of higher baseline hematoma volumes and high risk of hemorrhage [29]. Serum calcium has direct participation in platelet function and in several steps of the coagulation cascade [30]. Therefore, patients with low serum calcium might have impaired hemostatic mechanisms. And one study suggested that the occurrence of pulmonary edema in COVID-19 patients may be related to the radio-permeable TRPV4 channel, and the pulmonary edema caused by the virus can be alleviated by TRPV4 inhibitors [31]. In one case–cohort research, serum Ca was proved to be inversely associated with the risk of ischemic stroke [32]. So we acknowledge that the low serum calcium may also be one of the auxiliary mechanisms of multiple organ damage. Whether correcting calcium imbalance by taking a daily calcium supplement can avoid organ injury in the early stage of patients with mild/moderate COVID-19 needs future clinical research.

In conclusion, low calcium is not only the electrolyte imbalance caused by viral infection, but also one of the key factors causing multiorgan injury, and it may help to judge the severity of disease in the early stage of virus. Therefore, low calcium is a new target of concern for COVID-19.

## Supplementary Material

Supplementary Tables S1-S2

## Data Availability

All data included in the present study are available upon request by contact with the corresponding author.

## Abbreviations

ACE2: angiotensin-converting enzyme 2
ALT: alanine aminotransferase
AST: aspartate aminotransferase
BUN: blood urea nitrogen
Ca: Calcium
COVID-19: coronavirus disease 2019
CRE: creatinine
cTnI: Cardiac Troponin I
DBIL: direct bilirubin
eGFR: estimated glomerular filtration rate
hs-cTnI: highly sensitive cTnI
IL: interleukin
IRB: Institutional Review Board
NT-proBNP: N-terminal pro b-type natriuretic peptide
PT: prothrombin time
RT-PCR: real-time polymerase chain reaction
SARS-CoV-2: severe acute respiratory syndrome coronavirus 2
TBIL: total bilirubin
WMD: Weighted Mean Difference
95% CI: 95% confidence interval
