# Supplementary material for: Low serum calcium: a new, important indicator of COVID-19 patients from mild/moderate to severe/critical
Source: Biosci Rep. 2020 Dec 22;40(12):BSR20202690. doi: 10.1042/BSR20202690 (PMC7755121; doi:10.1042/BSR20202690)
Supplement: Supplementary Tables S1-S2 [file BSR-2020-2690_supp.pdf]

Supplement Table 1. Serological indicators of multiple organ injury in patients stratified by serum calcium

	Normal Ca	Low Ca	P Value
**N**	54	73	
**hs-cTnI (>15.6pg/mL)**	3/54 (6.3%)	23/73 (34.9%)	3.39E-04
**NT-proBNP (>169pg/mL)**	10/54 (20.0%)	35/73 (53.9%)	6.90E-05
**BUN (>7.5mmol/L)**	2/54 (3.7%)	15/73 (20.6%)	0.006
**CRE (>84umol/L)**	8/54 (14.8%)	21/73 (28.8%)	0.064
**eGFR (<90ml/min/1.73)**	14/54 (26.0%)	44/73 (60.3%)	1.22E-04
**ALT (>41U/L)**	14/54 (25.9%)	22/73 (30.1%)	0.603
**AST (>40U/L)**	9/54 (16.7%)	29/73 (39.7%)	0.005
**TBIL (>26umol/L)**	0/54 (0.0%)	6/73 (8.2%)	0.038[#]
**DBIL (>8umol/L)**	2/54 (3.7%)	14/73 (19.2%)	0.009
**PT (>14.5s)**	7/54 (13.5%)	32/73 (45.1%)	1.92E-04
**SpO_2 (mean±S.D.)**	96.7±2.3	92.6±8.4	1.44E-04

Supplement Table 2. Immune cytokines level in blood in patients stratified by serum calcium

	Normal Ca	Low Ca	P Value
**N**	36	57	
**IL-1β (>5pg/mL)**	3/36 (8.3%)	5/57 (8.8%)	>0.05
**IL-2R (>710U/mL)**	4/36 (11.1%)	22/57 (38.6%)	0.004
**IL-6 (>7 pg/mL)**	3/36 (8.3%)	31/57 (54.4%)	7.0E-06
**IL-8 (>62 pg/mL)**	0/36 (0.0%)	7/57 (12.3%)	0.041
**IL-10 (>9.1 pg/mL)**	3/36 (8.3%)	18/57 (31.6%)	0.009
**TNF-á(>8.1 pg/mL)**	11/36 (30.6%)	26/57 (45.6%)	>0.05
